# Short-term-outcomes of idiopathic epiretinal membranes treated with pars-plana-vitrectomy – examination of visual function and OCT-morphology

**DOI:** 10.1186/s40942-023-00496-3

**Published:** 2023-09-14

**Authors:** Verena Anna Englmaier, Jens Julian Storp, Nicole Eter, Sami Al-Nawaiseh

**Affiliations:** Department of Ophthalmology, University Medical Center Muenster, University Hospital Muenster, Albert-Schweitzer-Campus 1, Building D15, 48149 Muenster, Germany

**Keywords:** Gliosis, Oct, Optical coherence tomography, Retina, Classification, Biomarkers, Staging

## Abstract

**Background:**

Epiretinal membranes (ERM) represent one of the most common findings in retinal examination. Structural changes of the retinal layers in patients with ERM can be visualized and classified using OCT. The purpose of this study is to evaluate structural and functional changes related to surgical treatment of ERM.

**Methods:**

Monocentric retrospective analysis of 92 patients who underwent 23-gauge-pars plana vitrectomy (ppV) combined with cataract surgery for idiopathic ERM from 2015 to 2020. Visual acuity was determined directly preoperatively, at four weeks and three months postoperatively. Disease stage and tomographic biomarkers related to ERM were assessed in OCT imaging.

**Results:**

92 eyes of 92 patients were included. At the time of surgery, the mean patient age was 71 years. Visual acuity improved significantly by 2 lines postoperatively, on average from LogMar 0.4 to 0.2 (p < 0.001). Disease stage regressed from stage 3 to stage 2 postoperatively (p < 0.001). No patient had stage 4 postoperatively (n = 0). In the presence of preoperative intraretinal fluid, mean retinal thickness was 488 μm and decreased to 392 μm postoperatively (n = 32; p < 0.001). Preoperative presence of a Cotton Ball Sign (n = 30) was associated with better visual acuity (p = 0.009). This was also visible in patients with preoperative vitreomacular traction syndrome (p < 0.001). The presence of preoperative intraretinal fluid showed a tendency towards better disease staging after surgery (p = 0.080).

**Conclusion:**

Surgery was able to achieve visual improvement and morphological regression of the preoperative OCT findings related to ERM. ppV led to a reduction in retinal thickness and disease stage. The presence of the Cotton Ball Sign and vitreomacular traction was associated with better visual acuity in the follow-up period. In our cohort the preoperative presence of intraretinal fluid showed a tendency for better postoperative disease staging.

## Introduction

Idiopathic epiretinal membranes (ERM) are one of the most prevalent clinical findings, and their frequency increases with age [1]. Idiopathic ERM is thought to be caused by a pathological detachment of the hyaloid body [2, 3] which is accompanied by a proliferation of myofibroblasts, Mueller-Glia cells, hyalocytes, and retinal pigment epithelium (RPE) supported by growth hormones and cytokines [1, 2].

As ERM progress, they can cause visual impairment for the patient in the form of aniseikonia and metamorphopsia. Clinicians may find a discrepancy between clinical findings and the visual and overall subjective impairment of patients [4–6].

With the advancement of new diagnostic technologies, imaging can be used to detect ERM. Govetto et al. proposed a novel grading system that employs optical coherence tomography (OCT) to classify ERM into four groups [7].

They discovered that certain changes can be predictive [8] and can aid the postoperative prognosis [9, 10].

**Fig. 1:**
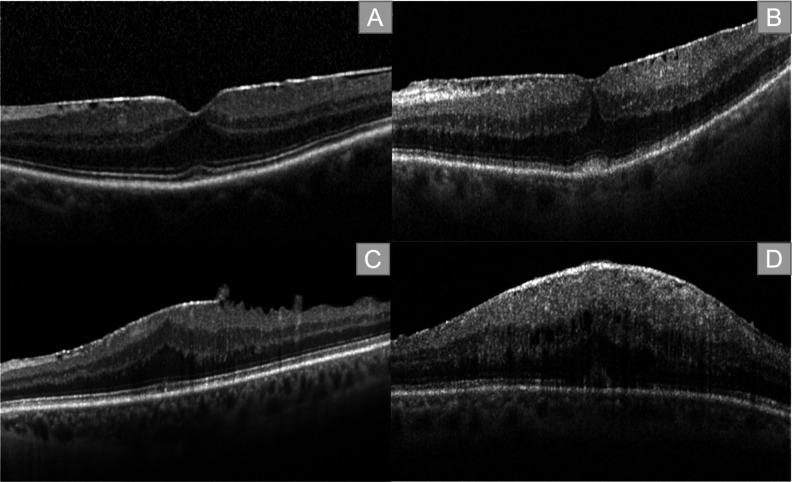
Staging of epiretinal membranes (ERM) according to Govetto et al. **A**: Stage 1: presence of foveal pit, retinal layers are defined. **B**: Stage 2: Absence of clear foveal pit, retinal layers are defined. **C**: Stage 3: Absence of foveal pit, retinal leyers are defined, ectopic inner foveal layers (EIFL). **D**: Stage 4: Absence of foveal pit, retinal layers are disrupted, presence of EIFL

**Fig. 2:**
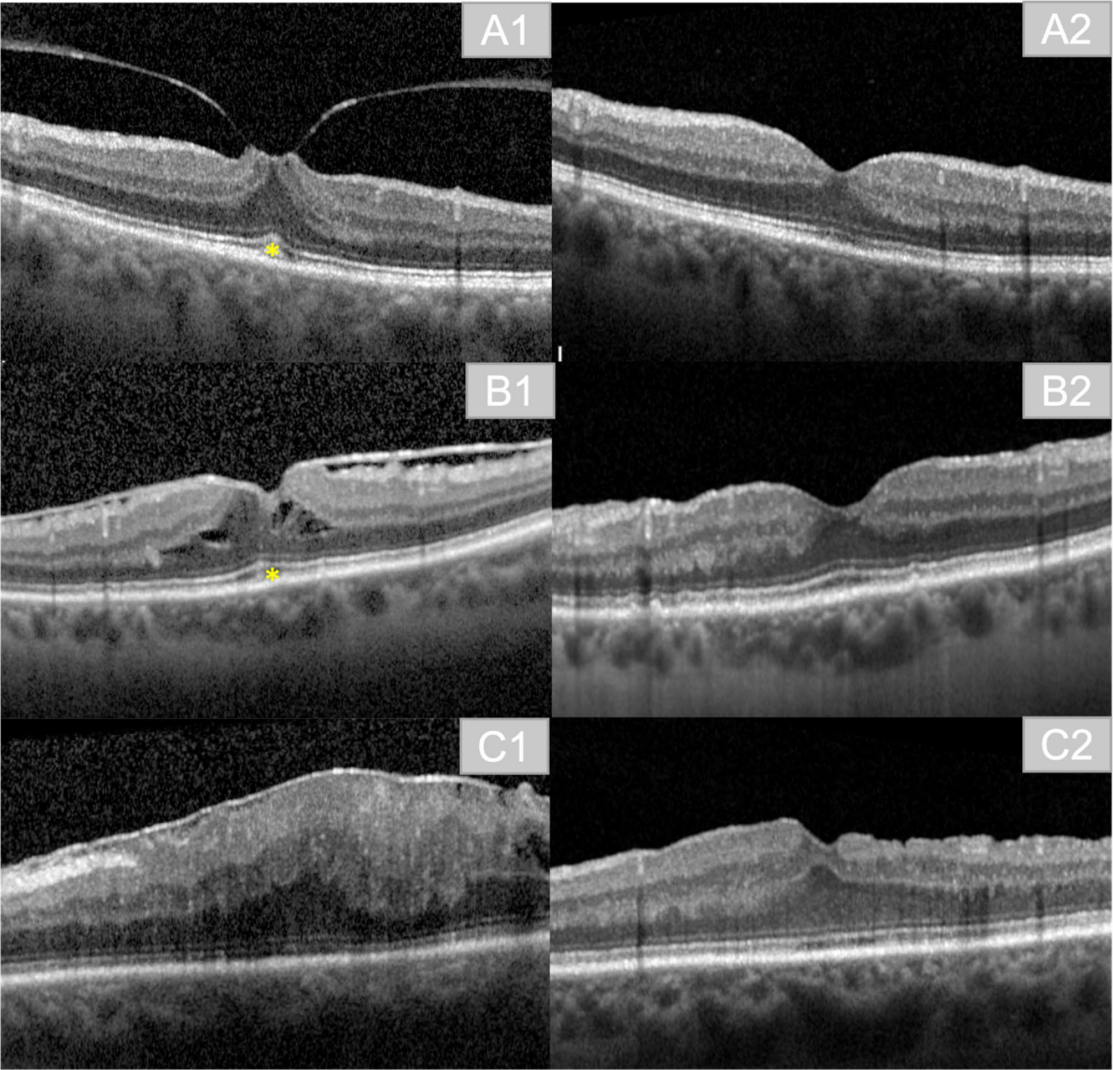
Exemplary imaging of pre- and postoperative results. **A1**: preoperative finding in a patient with vitreomacular traction (VMT) and Cotton Ball sign (*). **A2**: postoperative result in the same patient at three months postoperatively. **B1**: preoperative finding in a patient with ERM, Cotton Ball sign (*), and intraretinal cavities due to ERM. **B2**: postoperative result in the same patient at three months postoperatively. **C1**: preoperative finding in a patient with ERM Stage 4. **C2**: postoperative result in the same patient at three months postoperatively

The persistence of a foveal pit and physiological differentiation between retinal layers correspond with the first stage in the classification of Govetto et al. Discrimination between layers is still possible in the second stage, despite the absence of the foveal pit. The disappearance of the foveal pit and the presence of ectopic inner foveal layers (EIFL) characterize stage three. In stage four the foveal pit is gone, the retinal layers are disturbed and EIFL are present (Figs. 1A-D and 2C1) [7, 8].

A preoperatively increased central foveal thickness (CFT) is another OCT finding that stands out in clinical examination in patients with ERM. A decrease in CFT can be achieved through surgical removal of the ERM [2, 4, 5]. It is unknown, whether there is an association between preoperative CFT and postoperative visual acuity [11].

Increased CFT correlates with tractional forces and changes in the foveal contour caused by ERM [9, 12], either through vitreomacular traction, macular edema, or pseudolamellar morphologies. Some publications further classify ERM according to the presence of vitreomacular traction (VMT) (Fig. 2A1, **A2**), global adhesion (GA), or partial adhesion (PA) [13].

Furthermore, tractional forces of ERMs can cause a distortion of the inner retinal layers, resulting in EIFL [14]. At the level or the outer retina, traction can lead to the formation of a central bouquet (CBA), commonly referred to as Cotton Ball sign (Fig. 2, A1, B1). ERM can also cause further disruption to the layers of the outer retina, such as the Interdigitation Zone (IZ) and the Ellipsoid Zone (EZ). Disruption of IZ and EZ is associated with poorer BCVA after ERM surgery [10, 15, 16].

The removal of the membrane using pars-plana Vitrectomy (ppV) combined with inner limiting membrane (ILM) peeling remains the gold standard for treating ERM.

This study aims to investigate the structural and functional rehabilitation in eyes that received ppV combined with ERM and ILM peeling. Its main outcome lies in the evaluation of various ERM-related OCT-changes and –biomarkers. Biomarkers that were included are: presence of intraretinal fluid prior to surgery, preoperative and postoperative CFT, presence of CBA preoperatively, disruption of the EZ and IZ and preoperative presence of VMT.

We aimed to investigate the association between the abovementioned OCT-biomarkers and the postoperative outcome, which was based on BCVA and disease stage over a follow-up period of three months.

## Methods

### Patients and diagnostic modalities

This retrospective, monocentric study compiled with the standards issued by the local ethics committee of the Medical Association of Westfalen-Lippe and the University of Münster, Germany and adhered to the tenants of the Declaration of Helsinki.

The retrospective analysis included data from 01/2015 to 12/2020, acquired at the Department of Ophthalmology at the University Medical Center Hospital of Münster, Germany.

Data were retrieved from electronic patient files compiled in the patient management systems Orbis (Dedalus Healthcare GmbH, Bonn) and FIDUS (Arztservice Wente GmbH, Darmstadt), using the OPS (operations and procedures)-codes “5-158.22”, “5-158.23”, “5-158,25” and “5-158.26” for ppV with cataract surgery in combination with ICD (International classification of diseases)-10 codes “H35.3”, “H35.38” for ERM. This initial query showed a total of 483 patients. All included patients underwent 23-gauge-ppV for ERM peeling combined with cataract surgery. All patients were phakic at the time of the surgery.

Intraoperatively the trocars were positioned, afterwards a core vitrectomy was performed and a posterior vitreous detachment was induced, if not already present. For better visualization of the ERM, the ERM and ILM were dyed using indocyanine green (ICG; concentration 1 mg/ml) prior to the peeling. After peeling, the patients received an endotamponade, which was in all patients gas (C2F6; 18%). The surgery was always performed by the same three surgeons.

In some cases mild complications like postoperative hypotony, hypertony or fibrin reactions were visible, which resolved under topic therapy within the first postoperative week. These mild complications did not lead to an exclusion from the analysis.

If media opacities (e.g. mature cataract) prevented high quality imaging, patients were not eligible for inclusion. Patients with age related macular degeneration, macular hole, diabetic retinopathy, glaucoma, trauma, amblyopia, or uveitis were not eligible for inclusion. Patients who had undergone previous vitreoretinal operations, previous cataract surgery or developed macular edema after ERM operation were excluded as well. Cases requiring subsequent surgery during the follow-up period were also excluded.

The application of these criteria reduced the total number of eligible patients to 92. If patients had undergone surgeries on both eyes, one eye was chosen randomly.

Data on best corrected visual acuity (BCVA) as well as OCT data of the central macula one day prior to the operation were drawn into the study as baseline values for each patient. Patients were scheduled to re-visit with the clinic 4 weeks after the operation, as well as at 3 months postoperatively. Data from each follow-up visit were included in this study.

BCVA is reported as logarithm of the minimum angle of resolution (LogMAR).

OCT imaging was performed using the Spectralis ® spectral domain-OCT (Heidelberg Engineering GmbH, Heidelberg, Germany). Disease severity was determined preoperatively and at each visit postoperatively in accordance with the OCT based classification system by Govetto et al. [7]. Even though the classification proposed by Govetto et al. is used in presence of ERM, the structural features defining the classification, such as EIFL, remain differentiatable postoperatively. Therefore, we applied the system by Govetto et al. for both pre- and postoperative OCT images.The classification encompassed the following stages for pre- and postoperative OCT images [7]:

Stage 1: foveal depression is visible, distinguishable retinal layers.

Stage 2: no foveal depression is visible, retinal layers are still distinguishable.

Stage 3: presence of EIFL, different retinal layers are still visible.

Stage 4: presence of EIFL, distortion of retinal layers.

Apart from the classification, we looked for other specific OCT-biomarkers, including preoperative intraretinal fluid, CBA, integrity of the EZ, IZ and vitreomacular traction.

We evaluated the CFT both before and after surgery. CFT was determined using the Heidelberg Spectralis ® spectral domain-OCT. As a result, any decrease or increase in thickness was precisely reported. The average CFT for our patient group with a mean age of 71 is in line with results presented by Meyer et al. 270.17 μm [17].

Two specialists worked independently to interpret the OCT biomarkers. If the two could not agree on the interpretation of the OCT data, a third expert was consulted.

### Statistical analysis

Data were recorded in the spreadsheet software Microsoft Office Excel (Microsoft, Redmond, WA, USA) (2010). Statistical analysis was performed using SPSS (IBM SPSS Statistics 28).

As the normal distribution assumption could not be ensured, Mann-Whitney-U-Test was used to compare preoperative and postoperative results. We report p-values falling below 0.05 as significant findings. After the first analysis using Mann-Whitney-U-Test, we performed a linear regression analysis to compare the different biomarkers with their possible postoperative outcome regarding BCVA and disease staging.

Due to the purely exploratory purpose of this study, no adjustment for multiple testing was made. The findings presented should thus be treated with care or confirmed in a separate study. To account for a possible bias related to the extent of each OCT biomarker, biomarker-dependent subgroups were formed.

We acknowledge support from the Institute for Biostatistics and Clinical Research of the University Hospital Münster, Germany.

## Results

We included 92 eyes of 92 patients.

Baseline patient characteristics are shown in Table 1. The follow-up time was 91 days (three months). Table 2 shows the mean results in BCVA and disease staging for the study population.

**Table 1:** Baseline characteristics of the study population. Values are reported as absolute and relative (%) frequencies where applicable. Every OCT-biomarker is displayed in Table 1. Characteristics of each subgroup according to disease stage is displayed

Parameter		preoperative	postoperative
Patients, n	92	92	92
eyes, n (%)	92	92	92
right	45 (49%)	45 (49%)	45 (49%)
left	47 (51%)	47 (51%)	47 (51%)
gender, n (%)			
female	53 (58%)	53 (58%)	53 (58%)
male	39 (42%)	39 (42%)	39 (42%)
Age*, y			
Mean ± SD	71.08 ± 7.31		
Median	71		
Range	50–84		
BCVA (LogMar)		0.4	0.2
Disease Stages (median)		3	2
Intraretinal fluid, n		32	5
Retinal Thickness (µm)		488	392
Disease Stage 1 (n)		5	12
Disease Stage 2 (n)		17	12
Disease Stage 3 (n)		6	8
Disease Stage 4 (n)		4	0
CFT (µm)		446.5	383µm
Disease Stage 1 (n)		6	16
Disease Stage 2 (n)		22	30
Disease Stage 3 (n)		36	19
Disease Stage 4 (n)		0	0
CBA, n		30	11
Disease Stage 1 (n)		1	5
Disease Stage 2 (n)		10	15
Disease Stage 3 (n)		19	9
Disease Stage 4 (n)		0	0
EZ/IZ disruption, n		39	25
Disease Stage 1 (n)		1	9
Disease Stage 2 (n)		9	16
Disease Stage 3 (n)		24	14
Disease Stage 4 (n)		5	0
VMT, n		14	0
Disease Stage 1 (n)		1	5
Disease Stage 2 (n)		4	7
Disease Stage 3 (n)		7	2
Disease Stage 4 (n)		2	0

n = number, y = years, *at time of surgery; LogMar = logarithm of the minimum angle of resolution, SD = standard deviation; BCVA = best corrected visual acuity; CFT = central foveal thickness; CBA = Anomaly of the central bouquet; EZ = ellipsoid zone; IZ = interdigitation zone; VMT = vitreomacular traction

**Table 2:** Baseline data of all included patients for BCVA and disease stages; BCVA values are presented as median (25% quartile; 75% quartile); p-values under 0.05 were considered statistically significant

Parameter	preoperative	postoperative	P - value
BCVA (LogMar)	0.4 (0.3; 0,5)	0.2 (0.1; 0.4)	**< 0.001**
Disease Stages	3 (2; 3)	2 (2; 3)	**< 0.001**
Disease Stage 1 (n)	6	21	
Disease Stage 2 (n)	23	37	
Disease Stage 3 (n)	49	34	
Disease Stage 4 (n)	14	0	

*n = number; LogMar = logarithm of the minimum angle of resolution, BCVA = best corrected visual acuity*

### Visual acuity

The mean BCVA (LogMar) was 0.47 before and 0.3 after surgery (p < 0.001). The median BCVA improved from 0.4 to 0.2 (p < 0.001) (Table 2).

### Disease stages

When we compared the pre- and postoperative results of disease severity determined in accordance with the Govetto staging as mentioned above, we found statistically significant improvement after surgery (p < 0.001). Preoperatively the mean disease severity was stage 3, which improved morphologically to stage 2 after surgery (Table 2).

Preoperative stage 4 could be found in 14 patients. Postoperatively, no patient presented with stage 4.

### Morphological criteria

At baseline 32 patients had intraretinal fluid, 30 presented CBA and 39 showed a disruption of the EZ/IZ integrity. Among all patients, 14 presented with vitreomacular traction. In accordance with these morphological findings the following subgroups were assembled (Table 3):

**Table 3:** morphological criteria, preoperative and postoperative mean BCVA and disease staging; p-value under 0.05 was considered statistically significant (Significant values are bold). *: p-Value after comparative analysis using Mann-Whitney-U-Test; **: p-Value after regression analysis with linear regression

Morphological criteria	Number (n)	preoperative BCVA (LogMar; mean)	postoperative BCVA (LogMar; mean)	p-Value*	p-Value **	preoperative Disease staging (median)	postoperative Disease staging (median)	p-Value *	p-Value **
Intraretinal fluid	32	0.5	0.3	**< 0.001**	0.283	3	2	**< 0.001**	0.080
Preoperative increased CFT	65	0.4	0.2	**< 0.001**	0.104	3	2	**< 0.001**	0.258
Cotton Ball Sign/ CBA	30	0.5	0.3	**0.03**	**0.009**	3	2	**< 0.001**	0.120
Disrupted EZ/IZ Integrity	39	0.4	0.3	0.93	0.949	3	2	0.41	0.131
VMT	14	0.5	0.3	**0.003**	**< 0.001**	2	1	0.28	0.533

*n: Number; BCVA: best corrected visual acuity; LogMar = logarithm of the minimum angle of resolution; preop. = preoperatively; CFT: central foveal thickness; CBA: Anomaly of the central bouquet; EZ: ellipsoid zone; IZ: Interdigitation Zone; VMT: vitreomacular traction*

#### Intraretinal fluid

Intraretinal fluid was present in 32 patients preoperatively. The decrease in retinal thickness from 488 μm to 392 μm was statistically significant (p < 0.001). When we compared the BCVA evolution in these patients, they showed significantly better BCVA and disease stage postoperatively when we applied Mann-Whitney-U-Test (each: p < 0,001).

Regression analysis showed no statistically significant improvement in BCVA (p = 0.283) postoperatively and only a trend towards statistical significance for better disease staging (p = 0.08) (Table 3).

#### CFT

At baseline most patients represented a CFT above the average (> 270.71 age-matched [17]), which decreased significantly postoperatively (p = 0.004).

The mean preoperative CFT was 446.5 μm and decreased to 383 μm. In these patients, better BCVA postoperatively could be reached and showed a significantly lower OCT stage after surgery (each: p < 0.001) using comparative testing with Mann-Whitney-U-Test.

These results could not be confirmed by regression analysis, as both showed no statistical significant improvement. BCVA improvement presented a slight trend towards statistical significance (p = 0.104); disease staging did not improve significantly (p = 0.258) (Table 3).

#### CBA

Preoperative CBA was detected in 30 patients. This patient population showed better postoperative stages (p < 0.001) and BCVA (p = 0.03). Regression analysis confirmed the improvement in BCVA postoperatively (p = 0.009) (Table 3).

Postoperatively CBA resolved in 19 patients (63%). The remaining 11 patients presented with a Cotton Ball sign at the postoperative control visits (37%).

#### EZ/IZ integrity

Patients with EZ and IZ disruption were subsumed under one group as the layers could not always be separated. There were neither statistically significant changes in BCVA in the group with disruption (p = 0.928), nor a statistically significant improvement of the postoperative disease stage (p = 0.407). Regression analysis confirmed the results of Mann-Whitney-U-Test for the EZ/IZ Integrity (BCVA: p = 0.949; Disease staging: p = 0.131) (Table 3).

#### Vitreomacular traction (VMT)

Patients with preoperative VMT had significantly better postoperative BCVA (p = 0.003) but showed no significant differences regarding the postoperative disease stage (p = 0.28). Similar results were achieved when we performed a regression analysis in those patients (BCVA: p < 0.001; Disease staging: p = 0.533) (Table 3).

### Interpretation of the data

The two expert examiners reached agreement in 87 of 92 patients.

In 5 patients, a third expert was consulted to determine the disease stage and biomarkers.

## Discussion

Our findings are widely consistent with earlier research that looked at patients’ visual acuity and disease stage results following vitrectomy for ERM. Overall, we saw an improvement in BCVA and disease stage after surgery.

BCVA improvement probably results from the reduction of metamorphopsia and the fact that the procedure was performed in combination with a cataract removal and intraocular lens implantation at our clinic. Other studies show that both are likely contributors to the better postoperative BCVA [18–20]. Reduction of metamorphopsia can be achieved with ppV combined with ERM and ILM peeling. ILM, being the basal membrane of the Mueller glia cells, acts as a leading structure for ectopic cells to grow on, thereby supporting ERM formation. Combined ERM and ILM peeling leads to a smaller chance of reoccurrence of ERM [21, 22] and an overall reduction of metamorphopsia.

When we examined the vitreoretinal interface, we found that our VMT patient cohort frequently had extrafoveal attachment of the ERM. The extrafoveal traction may have led to reduced stresses on the central retina, which could account for the lower preoperative staging of stage 2.

Although we were unable to demonstrate a substantial improvement in staging, our cohort showed an improvement in BCVA after surgery. Thereby, our findings are consistent with earlier studies that have shown an increase in BCVA following ppV [23, 24].

At the level of the inner retinal layers, tractional forces can lead to EIFL and an increased CFT. Govetto et al. found, that EIFL are associated with a decrease in BCVA, and overall reduced surgical outcome after vitrectomy [8].

Reduction of the tractional forces also leads to a drop in CFT, which showed statistically significant improvement in BCVA and staging in our patient group in the comparative statistical analysis. As regression analysis could not support the statistical significance, it highlights the inconsistency regarding the impact of preoperative and postoperative CFT on the outcome after ERM-peeling [10, 15, 25, 26]. While Itoh et al. could not detect any correlation between the preoperative CFT and the postoperative BCVA [15], Park et al. were able to show a positive tendency but no statistical significance [25]. In contrast, there are other studies that support a positive correlation for decrease of CFT and BCVA after 6 months [26].

Besides the increase in CFT, strong traction of ERM can lead to fluid accumulation [26]. The current literature supports the positive effect of ppV combined with peeling for a better postoperative BCVA in patients with intraretinal fluid prior to surgery [9, 27], which is in line with the results presented in this work.

A reduction of the CFT and the intraretinal fluid accumulation postoperatively may contribute to a reduction of metamorphopsia, which can be associated with a better postoperative BCVA [18–20].

At the level of the outer retina, we were able to show that disease stage and BCVA both improved significantly, if a CBA was present preoperatively. We were further able to demonstrate a improvement in staging using Mann-Whitney-U-Test, whereas regression analysis only showed a trend towards a postoperative better staging (Table 1**and** Table 3).

Similar conclusions were drawn by Govetto et al., who discovered that this biomarker was associated with a postoperative disease stage improvement [28, 29]. Additionally, they discovered a relation between the CBA and Govetto Stadium 2 [14], which we were not able to reproduce, as most of our patients with CBA showed stage 3 preoperatively.

Ortoli et al. demonstrated a postoperative reduction of the cotton ball sign in 97% of cases in their study population. With a resolved CBA after surgery of roughly 60% in other trials, the outcomes are comparable to those of our group [29, 30].

No statistically significant postoperative improvement in BCVA or staging was seen when EZ/IZ integrity was compromised. The discontinuity at the level of the photoreceptors could be held accountable for the overall reduced BCVA [15]. Some studies even describe worse postoperative visual function after longer-lasting breakdown of EZ/IZ integrity [15, 31], which fortunately we did not see in the three-month follow-up of our patient cohort.

The results of this study show that different biomarkers can provide insights into the potential postpoperative development. Especially in cases where tractional forces (intraretinal fluid, CBA, reduction of CFT and VMT) were present preoperatively, we were able to show a trend for statistically significant improvement in BCVA and partially in postoperative disease staging. This supports the recommendation to treat patients suffering from ERM with ppV combined with peeling of the ERM and the ILM.

### Limitations

This study was carried out retrospectively at a tertiary eye care center. Given the retrospective nature of this study and the inherent limitations posed to the sample size, our ability to provide insights regarding future estimates is constrained. The extent to which the presented trial findings persist or subside over an extended follow-up period remains uncertain. With a comparably short follow-up period of three months further prospective long-term follow-up studies are needed to better evaluate the morphological changes associated with the BCVA and disease staging.

Secondly, media opacities, such as those caused by a cataract prior to surgery, caused variations in OCT quality. Although poor image quality was a criterion for exclusion, the differentiation between smaller retinal structures and layers has sometimes been challenging. Further high-resolution OCT studies are required to evaluate the postoperative development in individual retinal layers.

Thirdly, the patients underwent combined ERM and cataract surgery. It is still unclear how much of the improvement in BCVA is attributable to the removal of ERM and cataract surgery, respectively.

## Conclusion

This study shows that ppV combined with ILM Peeling for ERM can achieve better disease staging and better postoperative BCVA directly after surgery and up to three months postoperatively.

This is in line with other research and underlines the benefit of ppV combined with ILM peeling and cataract surgery as a surgical treatment for ERM.

Furthermore, there are some OCT-findings that are associated with a significant improvement in disease stage and BCVA.

The presence of the Cotton Ball Sign and vitreomacular traction syndrome was associated with better postoperative BCVA in the follow-up period of three months.

Preoperative presence of intraretinal fluid showed a tendency for better postoperative disease staging.

## Data Availability

The datasets which were analysed during the current study are available from the corresponding author on reasonable request.
